# Health Impacts of Migration and Policy on the Iranian Diaspora

**DOI:** 10.1007/s10903-025-01844-1

**Published:** 2026-01-07

**Authors:** Jihye Lee, Mitra Naseh, Sahar Badiezadeh, Sarvenaz Taridashti, Erick da Luz Scherf

**Affiliations:** 1 Brown School, Washington University in St. Louis, St Louis, USA; 2 Global Governance & Human Security, University of Massachusetts Boston, Boston, USA; 3 Industrial & Organizational psychology, Montclair State University, Montclair, USA; 4 School of Social Work, University of Alabama, Tuscaloosa, USA

**Keywords:** Highly educated immigrants (HEIs), Migration policies, Iranian diaspora, Integration, Nationality-based policies

## Abstract

Migration is shaped by a complex interplay of political, economic, and social factors that drive individuals to seek stability and better prospects abroad. Since the 1979 Islamic Revolution, Iran has experienced significant emigration of its highly educated population, driven by political instability, economic challenges, and restrictive societal norms. This study explores how U.S. immigration policies affect the health and well-being of highly educated Iranian immigrants, with particular attention given to the impact of nationality-based restrictions on psychosocial outcomes. Using a phenomenological approach, we analyzed in-depth interviews with 40 participants to examine the lived experiences of highly educated Iranian immigrants in the United States. Three major themes emerged: (1) Migration motivations and psychological weight; (2) Institutional barriers and psychosocial strain; and (3) Economic hardship and health vulnerability. Despite advanced degrees and partial socioeconomic integration, participants faced persistent visa precarity, family separation, and financial exclusion, all contributing to increased psychosocial vulnerability. Our findings highlight how immigration policy acts as a social determinant of health, shaping emotional well-being, social connections, and economic stability. More inclusive immigration policies, such as decoupling immigration processes from geopolitical tensions and enhancing financial accessibility, are needed to enhance integration pathways for highly educated migrants and ultimately, ensure this population’s well-being.

**Supplementary Information:**

The online version contains supplementary material available at 10.1007/s10903-025-01844-1.

## Introduction

Migration is a multidimensional phenomenon shaped by intertwined social, political, and economic pressures that influence individuals’ decisions to relocate across borders [1]. The United States has long been a key destination for Iranian immigrants [2], though U.S. immigration policy has consistently sparked debate throughout different periods of history [3]. Among the U.S. immigrant population, Iranians form a distinctive group, with much of the migration driven by Iran’s political upheavals and socioeconomic challenges since the 1979 Islamic Revolution [4, 5]. This trend has continued, particularly among highly educated individuals seeking stability and opportunity abroad [3, 6].

The 1979 revolution, the Iran-Iraq War, and ongoing international sanctions marked the beginning of a large-scale Iranian diaspora, increasing the population abroad from an estimated half-million before the revolution to 5.4 million by 2023 [7], highlighting a continued pattern of global migration [8]. Educated Iranians were disproportionately affected by political repression and economic recession, resulting in significant outflows of professionals and intellectuals alienated by Iran’s shifting political environment [9]. Despite having high educational credentials – only 7% of Iranian migrants lack a high school diploma compared to 26% of the overall U.S. immigrant population [10] – these individuals face unique integration challenges, particularly due to diplomatic tensions between the U.S. and Iran [3].

Since the Islamic Revolution, U.S. immigration policies have imposed specific barriers for Iranian immigrants, including restrictive visa policies, prolonged security screenings, and limited consular access [11]. These restrictions create heightened challenges for highly educated Iranians, whose personal and professional pathways are shaped by the complex U.S.-Iran relationship. Research indicates that restrictive immigration policies targeting specific visa categories can generate unintended stressors, impacting both mental health and socioeconomic mobility [12, 13]. For example, nationality-specific bans and strict visa protocols, such as the Trump administration’s 2017 travel restrictions, disproportionately affected Iranian nationals and limited their access to opportunities [14]. These hurdles, driven by ongoing sociopolitical and economic challenges, have contributed to the “brain drain” that Iran continues to experience [15].

In this context, immigration policy operates as a social determinant of health, shaping how individuals navigate new environments, access services, and maintain emotional resilience [16]. For Iranian immigrants, the intersection of restrictive visa policies, racialized classification, and systemic exclusion has produced elevated psychosocial stress and unequal health outcomes [17]. While prior studies have examined Iranian migrants’ integration in countries such as Canada, Germany, and Sweden [18–25], limited research has explored how U.S. immigration policies affect the mental health and integration of highly educated Iranians.

This study addresses that gap by examining how both push-pull migration dynamics and U.S. immigration policies function as structural determinants of health. It considers not only the individual experiences of highly educated Iranian immigrants, but also the broader implications for host communities. Specifically, the study investigates how visa restrictions and racialized categorizations – such as the ongoing classification of Middle Eastern immigrants as “White” [26] – shape mental health, integration experiences, and socio-economic mobility among highly educated Iranian immigrants in the U.S.

### Conceptual Framework

The conceptual framework of the study is grounded in two intersecting theoretical frameworks: the push-pull theory of migration and the Social Determinants of Health (SDOH). The push-pull factor theory explains how migration decisions are shaped by opposing forces – “push” factors such as political repression, economic hardship, or social discrimination in the country of origin, and “pull” factors such as educational opportunities, political stability, or personal freedom in the destination country [27, 28]. While traditionally used to explain the mechanics of migration, this framework also helps illuminate the emotional and psychological consequences of prolonged exposure to instability, including chronic stress, fear of return, and the pressure to succeed abroad in the host society [29, 30].

Complementing this perspective, the SDOH framework examines how broader structural conditions – such as restrictive immigration policies, visa delays, financial exclusion, and family separation – influence migrants’ physical and mental health [16, 17, 31]. Rather than framing health solely through biological factors and/or access to healthcare, SDOH theory emphasizes the cumulative impact of social, economic, and legal environmental on well-being [17]. It enables an analysis of participants’ experiences – such as uncertainty, isolation, and constrained mobility – as symptoms of psychosocial strain rooted in structural exclusion [32, 33].

By integrating these frameworks, the study moves beyond individual narratives to investigate how migrants’ health, resilience, and integration trajectories are shaped by legal and social structures. Migration is thus understood not merely as a legal or economic event, but as a health-related and psychosocial phenomenon [34, 35]. This qualitative study leverages in-depth interviews to conduct a broader exploration of migration experiences in the U.S., focusing on the impact of restrictive migration policies on highly educated Iranians.

## Methods

### Ethical Considerations

The study was reviewed and approved by the Institutional Review Board of Portland State University (IRB Reference: 21725218) and Washington University in St. Louis (IRB Reference: 202403107). Verbal informed consent was initially obtained from all participants during the screening process while scheduling interviews, and again at the beginning of each interview. Participants were advised not to share personally identifiable information during the audio-recorded interviews. Audio recordings, only accessible by the research team, were permanently deleted upon completion of the transcription process.

### Participants

This study is a phenomenological study aiming to document the lived experiences of highly educated Iranian immigrants in the U.S., using a conceptual framework that examines their motivations for leaving Iran, their reasons for migrating to the U.S., and the role of immigration policies on social determinants of health. It draws from a subset of semi-structured interviews with 23 Iranian participants, taken from a broader phenomenological project examining the experiences of highly educated immigrants in the U.S. In addition, informed by the first part of the interviews, 17 more semi-structured interviews were conducted with other highly educated Iranian immigrants, bringing the total sample for the study to 40 interviews. The first set of interviews was conducted in 2023, under IRB approval from Portland State University, and the second set was conducted in 2024 under IRB approval from Washington University, with members of the original research team involved in both phases.

### Data Collection

Participants were recruited using convenience and snowball sampling methods. Interviews were in English via Zoom and audio recorded, with an average interview length of 43 min (Interviews ranged from 25 to 75 min). Participants had the option to provide written consent either by completing an electronic form or verbally at the start of the interview.

The interview protocol included seven open-ended questions that invited participants to reflect on their pre- and post-immigration experiences and the health-related impacts of immigration policy. The interviews focused on three interconnected areas: (1) key drivers of migration; (2) the impact of U.S. immigration policies, including visa limitations and financial sanctions, on participants’ health and well-being; and (3) the coping strategies they employed to navigate these challenges, such as relying on peer networks and community support. The full interview protocol is available as a Supplementary file (Annex 1).

### Analysis

The 40 recorded interviews were transcribed using Zoom’s transcription features, with transcripts subsequently reviewed by the research team to verify the accuracy, incorporate needed edits, and remove any personally identifiable information. Data was analyzed using reflexive thematic analysis, following Braun and Clarke’s approach [36–38]. This approach was employed to systematically identify, organize, and interpret themes within the data set [39]. The team then analyzed the identified themes and observations in consultation with the interviewers. To minimize bias, two team members, the first and fourth authors, independently coded a subset of five transcripts using open coding. Then, the second author reviewed and consolidated similar codes, developing a comprehensive codebook that guided the coding of the remaining transcripts. Reoccurring codes were iteratively reviewed by the research team to highlight emerging themes. Systematic consensus-building sessions among the authors ensured consistency and depth in the interpretation process [40]. Three main themes were selected for reporting in this manuscript: (1) Migration motivations and psychological weight; (2) Institutional barriers and psychosocial strain; and (3) Economic hardship and health vulnerability.

### Participant Characteristics

The sample consisted of 40 participants (Table 1), with a slightly higher proportion identifying as female (52.5%) compared to male (47.5%). Participants ranged in age from 28 to 36 years, with a mean age of 32.3. Most held or were pursuing doctoral degrees (72.5%), while the remaining 27.5% were enrolled in or had completed master’s programs. Participants had lived in the U.S. for an average of 7.2 years, with durations ranging from 2 to 18 years. Nearly all initially entered the U.S. on student visas (F-1, 97.5%), the majority of whom held single-entry visas (67.5%).

**Table 1:** Demographic characteristics^1^

Characteristic	Category	%
Age	28–36 (M = 32.3)	-
Gender	Male	19 (47.5%)
	Female	21 (52.5%)
Marital status	Married	10 (47.6%)
	Not Married	11 (52.4%)
Education level	Master Degree	11 (27.5%)
	Doctoral Degree	29 (72.5%)
Initial Visa Category	Student Visa (F-1)	39 (97.5%)
	Work Visa (H-1B)	1 (2.5%)
Initial Visa Entry Type	Single Entry Visa (1 years)	27 (67.5%)
	Multiple Entry Visa (2 years)	13 (32.5%)
Current visa Category	Student Visa (F-1, OPT, CPT)	11 (27.5%)
	Applying for Permanent Residency	16 (40%)
	Permanent Resident	12 (30%)
	Work Visa (H-1B)	1 (2.5%)
Years of Residency in the U.S	< 5	8 (22.2%)
(M = 7.2)	5–10	20 (55.6%)
	> 10	8 (22.2%)
Current Employment Status	Student	21 (80.8%)
	Academic (e.g., assistant professor)	3 (11.5%)
	Health professions (e.g., pharmacist)	2 (7.7%)
Income Level (Annual)	<$25,000	3 (12.5%)
	$25,000–$50,000	7 (29.2%)
	$50,000–$100,000	9 (37.5%)
	$100,000–$200,000	4 (16.7%)
	>$200,000	1 (4.2%)

OPT = Optional Practical Training; CPT = Curricular Practical Training; H-1B = Temporary Work Visa. ^1^Percentages represent proportions within each responding subgroup.

At the time of the interviews, participants’ immigration statuses varied: 27.5% remained on student visas (F-1) with Optional Practical Training (OPT) or Curricular Practical Training (CPT); 30% had obtained permanent residency; 40% were in the process of applying for permanent residency; and 2.5% held temporary work visas (H-1B). In terms of employment, 80.8% of participants were current students, while 19.2% were employed in academic roles (e.g., assistant professor) or health professions (e.g., pharmacy). Annual income levels also varied. The largest proportion reported annual earnings between $50,000 and $100,000 (37.5%), followed by those earning $25,000 to $50,000 (29.2%). Smaller segments reported income in the ranges of $100,000 to $200,000 (16.7%), less than $25,000 (12.5%), and above $200,000 (4.2%).

## Results

### Migration Motivations and Psychological Weight

Participants described a range of reasons for migrating from Iran to the U.S., shaped by intersecting concerns related to education, career development, political repression, and gender-based restrictions. These were not isolated decisions but responses to prolonged emotional strain and psychosocial stressors in Iran. Migration was often framed as a pathway not only to opportunity but also to psychological safety, autonomy, and survival. Many also spoke about the emotional toll of these conditions, describing feelings of fatigue, frustration, and anxiety contributed to their decision to leave.

Migration was frequently framed as a path to better academic and professional growth. A married female doctoral student with permanent residency in the U.S. mentioned: “I’m happy that I’m here because first I’m learning. Second, it’s a good opportunity for me too like experience and other education… which is… much more acceptable. And third that I’m living in the States. It’s a totally different country from my home country. More free for me. [IA-2]” A female healthcare professional with a doctoral degree in Public Health shared: “The first motivator was pursuing my education…and have more freedom and authority. [IA-17]”

Participants often framed migration not only as an opportunity abroad, but as a response to systemic limitations they faced in Iran. One doctoral student reflected on her broader dissatisfaction: “My immigration reason is not just because I really love research and a study…… the situation there is not good. [IA-3]” A female doctoral student with permanent residency expressed: “I feel homesick for my family from my home country for everything, but this is … the choice. We cannot go back because you know the situation is not good [in Iran]…Getting the visa for master degree is not easy. That’s why I should apply for Ph.D. [IA-12]”

Several participants shared how political instability and repression influenced their decision to leave. These experiences were often accompanied by fear, stress, and uncertainty. A female doctoral student with a student visa emphasized: “It was because of the freedom situation in Iran… I have freedoms that I haven’t in my own country. [IA-11]” A male participant on a student visa also explained:

Coming from Iran as a country where…your existence is a function of your state’s existence. Your view [is] free to the extent that your government wants you to be free…It was very stressful because all I had done. After the Green movement in Iran that I was one of the leaders in the demonstrations… I had a lot of friends jailed [and] arrested. I was one of the lucky ones who escaped. And after that I just decided to come to United States… I cannot live in a country like Iran anymore… That’s the right word, I fled Iran [because of] the frustration that I had. [IA-38]

Gender-specific challenges in Iran shaped the migration decisions of several participants, particularly women. As one female master’s degree student described, “I came from a country… [that was] oppressive in terms of facing women. [IA-1]” These challenges not only shaped life opportunities but also generated emotional strain. Many women spoke about the psychological toll of Iran’s mandatory hijab rule and associated social norms. One female participant, a master’s degree holder, explained: “You have to be [a] Hijabi girl. [IA-16]”

These restrictions were described not only as legal obligations, but as daily sources of anxiety, fear, and a constant reminder of gendered control. One participant recalled:

We all have to put stupid scarves [in Iran] and we don’t even have to the right to choose for…what we want to wear or not. The last year that I was in Iran was…so scary because of the killing of a girl [for] hijab and it was so scary…So, I wanted [and] I knew that I’m going to go in to emigrate to [the] U.S. [IA-11]

Another female doctoral student on a student visa described the emotional weight of being unable to “tolerate that stuff, forces for Hijab, and all these things. [IA-3]”, while another participant with a master’s degree in Public Health expressed a sense of relief after migration, saying, “Here I’m free… at least [I] wear whatever I like. [IA-7]”

A master’s degree holder with permanent residency explained her emotional toll to envision a “good life” in Iran: “With all considering the political situation of Iran, …I cannot believe a good life in Iran [because of] all restrictions on. I’m [a] human being, especially [as a] women…. So I considered continuing my education…to live a happy life. [IA-9]” One participant explained how cumulative gendered discrimination - from hijab mandates to workplace bias - left her feeling “traumatized”:

50% of your community [meaning] your population [of women] cannot even live in that country in peace … regarding my belief system, my hijab…even in my working mind, even in my education environment … It always was traumatized. [IA-5]

For some, the inability to advance professionally due to gender barriers was more than just a career concern – it was a source of persistent frustration and emotional strain. A female doctoral student reflected on the “glass ceiling” she encountered in Iran: “I was not getting promotion…because I was a woman…You know it’s like a glass ceiling… They deny that there is any glass ceiling, but there is. [IA-10]”

Gender-based and social discrimination also affected participants outside Iran’s gender binary system. A doctoral student who identified as a gay man shared his fears about returning to Iran: “I’m a member of the LGBT community [and] a gay man…I do not want to go back to Iran because it’s not safe for me. So, there’s always the anxiety of that… it’s very worrying. [IA-32]” In contrast to the experiences of women and LGBTQ + individuals, male participants often framed migration as a choice rather than a necessity. For instance, one male doctoral student said: “As of discrimination against gender, as a boy, I had this superior hand. [IA-6]”

### Institutional Barriers and Psychosocial Strain

After arriving in the U.S., participants encountered a different set of stressors – primarily legal precarity, visa-related restrictions, and systemic financial exclusion. These experiences produced a state of “limbo,” marked by emotional isolation, inability to reunite with family, and perceived discrimination based on Iranian nationality. Such legal and financial constraints were not merely bureaucratic inconveniences; they functioned as chronic stressors with clear psychosocial consequences.

For Iranian immigrants, the process of coming to the U.S. often begins with significant logistical challenges, notably the lack of a U.S. embassy in Iran, requiring travel to third countries for visa applications. In this context, one of the interviewees told us:

Getting … an American visa for an Iranian… it’s really hard. It’s very expensive. It’s very uncertain …In Iran, you don’t have … American consulate, so, they have to go either to … Turkey, Armenia, or something [to] do their biometrics, and then go there again for take their like passports to get like the stamp…Imagine how much money, and like with the rates and the sanctions on Iran. Just it’s going to be an intense amount of money that not every person or every family has. [IA-30]

The journey itself is only the beginning; lengthy administrative processes often exacerbate the uncertainty and anxiety caused by it. A female participant on a student visa described: “I know people who … get accepted to best universities in the U.S., and they’ve been in the process of [visa] clearance for a year…They’re not just … [as] lucky as me to be here because I get cleared after two months, and that two months was the hardest time of my life. [IA-11]” Another interviewee who has been in the U.S. for three years as a student on a single-entry visa highlighted the emotional burden of getting an entry visa by saying, “[Waiting for your entry visa is like] being in limbo is the worst feeling in the world because you don’t know, you cannot plan for your future. You [are] just in waiting, which kills you. That’s … very bad because you don’t know what will happen. [IA-14]”

Once in the U.S., the restrictions tied to single-entry visas cast a mental health burden over daily life. One of the male participants who had a doctoral degree explained, “Since I’m from Iran, the visa I had was single-entry. So, I haven’t been able to travel outside [the] U.S. since I came here, which is going on seven years. [IA-32]” Such constraints, combined with the lingering effects of the travel ban, created an overwhelming sense of isolation.

A recurring sentiment among participants was a recognition of inequality among participants when comparing their experiences to those of other international students. One of the participants, who was an epidemiologist and had been in the U.S. for a decade, stated:

I have a classmate from Nepal. He [went] back to Nepal, … stayed two months with [his] family…. We cannot do that. And if I go back to visit my family, the chance of coming back to [the] United States would be 50% or 20%, or even [zero] percent… So, we prefer, and we sacrifice some stuff to stay here until … [getting] our Green Cards, hopefully, [to] be able to visit our family. [IA-37]

One female participant who was a permanent resident and had entered the U.S. in 2013 with a single-entry student visa also remarked: “I was feeling [like] that I’m present in a big and sometimes beautiful attractive prison. And I’m working and I’m paid as a prisoner, but I’m not allowed to go [to my home country, or] to even have a visitor. [IA-40]”

The emotional toll of these policies was most acutely felt in the context of family separation. For participants, the inability to visit loved ones, be present during critical moments, or have family members visit the U.S. was a source of profound distress. A female doctoral student on a single-entry visa shared: “There were times … I was crying because I couldn’t get back to see my mom during five years…I really disagree with this, and I hope that there might be a way to get away from this. [IA-11]” A male interviewee who applied for a green card at the time of the interview also recounted a painful family separation experience: “Last year, my uncle … passed away… I was so close to him… but I couldn’t be there at the hospital … It was so difficult for me at that time. I [had]… about maybe two months [of] serious depression at that time, but I couldn’t [go] back to my country because … it’s very tricky for Iranians to be applying for visa… So, I decided to stay in the U.S. [IA-22]” He added:

My mother misses me a lot…Every night my mother asked me … “When [do] you want to [come] back?” … [I told her] “I don’t know. I’m not sure … when I can get my Green Card.” … it’s [family separation] also affects my mental life… because it’s my mother, [she is] really important for me, and when she’s crying in front of me, that’s really bad for me. [IA-22]

Participants also described emotional burden of being subjected to discrimination due to their nationality. A female permanent resident interviewee who had entered the U.S. in 2013 stated, “I think our crime was just related to our ethnicity and our nationality…We are all human beings. We have the right to live as others, right? But the only issue that was like putting me into such a kind of burden with my nationality. [IA-40]” Another participant who has been in the U.S. for nine years and is currently in the process of obtaining permanent residency shared how the lengthy background checks, especially for Iranians, made them feel targeted, “I have specifically felt some stuff in terms of like background check just like getting the visa to come in here like they were super long. And you know, when I was getting my [state] ID, … that took way longer than it should. They were like, “Oh, it’s a like a random background check that we have to do,” and I was pretty sure it’s not a random. [IA-30]” In this context, a male doctoral student on a student visa expressed: “Always as an Iranian, every time you fly, there’s more checkpoints you need to do, there [are] more questions to answer, there’s always more restrictions over that … usually when they see you’re Iranian. [IA-6]”

### Economic Hardship and Health Vulnerability

Participants described how U.S. sanctions and nationality-based financial restrictions intensified both emotional and economic vulnerability. Difficulties accessing banking services, securing loans, or receiving financial support from Iran led to chronic stress, exclusion, and financial instability. Despite legal immigration status, many felt penalized and stigmatized solely for their nationality, often describing the process as frustrating and bewildering. These barriers were not only logistical but also deeply emotional, contributing to a persistent sense of marginalization and psychological strain during resettlement.

Several interviewees recounted instances of being denied the opportunity to open a bank account. A female interviewee who has been in the U.S. since 2018 shared, “Some banks …, [Bank’s Name], … at the time in 2018, … refuse to open [a] bank account because of my nationality. They [said]… we cannot. However, I was under [a] J1 visa. [IA-1]” Another female participant, who arrived in the U.S. as an international student and was a permanent resident at the time of the interview, described a similar experience, stating, “When I came to the US, … there were not many banks [that would] open an account for Iranian passport [holders] … I was here with the F1 [Student] visa, [Bank’s Name] rejected me a bank account. [IA-2]”

Participants referred to these experiences as “discouraging,” “disheartening,” and “confusing.” In this context, a male participant who arrived in the U.S. as an international student expressed, “Those things [being denied a bank account] are very discouraging, … because, like, I haven’t done anything… I basically escaped my own government, why am I being sanctioned? [IA-30]” Another international student, frustrated by restrictions on accessing the banking system, stated, “Why, why? I’m [a] regular people, I’m ordinary people. I’m not related to any governmental families or political [groups], … [but] sanctions impact the financial aspects of my life. [IA-17]” Another student described his experience with the banking system as “puzzling,” asking, “If we are in the same country and the rules and regulations are the same for everyone …, how come one bank can do it [open an account for Iranians], the other one cannot do it? [IA-4]”

A few participants also noted that, besides finding a bank “fine with opening accounts” for Iranians, banks may randomly and suddenly close Iranian accounts, causing them distress. One participant said, “I had … my account there for two and a half years, and all [of a] sudden they just say that they have to close your account, and they just close my account. [IA-15]” Another participant recounted his friend’s experience, noting, “One of my friends that has a bank account in [Bank’s name]; [Bank’s name] closed his account because he was Iranian. [IA-22]”

Scrutiny and periodic requirements for additional documentation were additional recurring distressing issues, with many participants expressing frustration at how they were treated. A male participant who has been in the U.S. since 2012 recounted that, after having an account with a bank for over two years, they contacted him with what he described as “stupid questions” about his income, stating, “They called me with some stupid questions about … how I get my income. I basically … said … look at the checks that they deposit. [IA-13]” Another participant, who was a permanent resident at the time of the interview, recounted, “Bank… sent me a message … and said, you should provide us like a list of documentation… so many documents … the tone of the email was so threatening… It’s insulting… I’m educated, and I’m respected… how could you treat me like that? [IA-2]” One participant shared a more extreme experience, in which mentioning Iran in a bank transaction led to the closure of her account and the account of the sender. She described that this incident was related to a fundraiser for an animal shelter in Iran to cover the cost of transporting a dog to the U.S. She explained:

It was two years ago. I did a very small fundraising, asking people … to pay for purchasing a ticket for one of these disabled dogs …[to] be transferred here. [A] lady, poor her, … she is Iranian, but she was born here, and she didn’t know a lot about these things, and in the [Payment’s Name] memo she wrote ‘Iran Ward’, and they just blocked my accounts and her accounts, both of us, for I think one month. And I had to … send back and forth emails explain[ing] the situation that this dog is going to be transferred here. The money doesn’t go over there [Iran], everything will be spent here. [IA-17]

Interviewees also referenced their limited access to loans and financial services as a barrier and emotional burden. One participant explained that private lenders offering loans to legal immigrants would not serve Iranians: “I reached out to them and … they [Organization’s Name] told me no, it doesn’t provide this service to students from Iran, Sudan, Afghanistan, and six countries in this list. [IA-1]” Another participant stated, “I tried once to get a loan. It was a company that they were designated to work with international students…My application was declined because of obviously my nationality; due to sanctions. [IA-8]”

Participants also indicated that they faced difficulties transferring funds to and from Iran due to sanctions. One explained, “We don’t have any financial transaction allowance between Iran and [the U.S.] like the rest of the world. [IA-39]” Another participant echoed this challenge, stating, “Because of the… political issues between Iran and the United States; you cannot have direct money exchanges here… I cannot receive any money from my family. [IA-15]” In this context, a participant said that Iranians are “aliens” in the U.S. and added “Iranians cannot transfer any fund directly or even indirectly from Iran. So, even our families couldn’t support us. [IA-40]”

## Discussion

While most participants migrated for educational and professional growth, their narratives reveal that restrictive immigration policy and U.S.-Iran tensions created chronic stressors that adversely impacted their emotional well-being, social relationships, and financial stability. Migration, for this population, is not only a path to opportunity – it is also shaped by prolonged legal, institutional, and psychological strain.

Participants – predominantly young adults (mean age: 32.3 years) with advanced degrees – represent a highly skilled segment of the Iranian diaspora. Most entered the U.S. on single-entry student visas and transitioned to a mix of temporary and permanent statuses over time. While many reported middle-income earnings, reflecting partial socio-economic integration, their experiences highlighted how legal and institutional exclusion limited their full integration, causing isolation and distress. This pattern reflects broader demographic trends: Iranian migrants often demonstrate higher educational attainment than other immigrant populations in the U.S., yet contend with complex and restrictive migration policies [10].

Our findings suggest that participants’ migration decisions were shaped by a combination of “push” and “pull” factors. While many cited educational and career opportunities as primary motivations, their narratives – particularly among women and LGBTQ + individuals – often reflected deeper emotional and psychological concerns. For these groups, migration was not only a pursuit of opportunity but a survival strategy to protect mental and emotional well-being. Chronic anxiety, gender-based repression, and threats to personal autonomy were described as direct triggers for emigration – conditions widely recognized as psychological stressors in politically repressive environments [41, 42]. State-enforced hijab laws, morality policing, and fears of persecution based on sexual identity were frequently mentioned, aligning with research on authoritarian gender governance, trauma, and structural vulnerability [43, 44]. These experiences also echo Chaichian’s [19] analysis of Iran’s “brain drain,” in which systemic exclusion pushes skilled individuals to seek freedom and opportunity abroad.

Yet arrival in the U.S. did not eliminate stress; it introduced new forms of exclusion tied to visa status and national origin. Participants described prolonged legal uncertainty stemming from single-entry visa restrictions, processing delays, and opaque security checks. These experiences – often described as “being in limbo” or a “beautiful attractive prison” – align with Menjívar and Abrego’s [45] concept of legal violence: the slow, bureaucratic production of harm through immigration systems. Visa precarity disrupted participants’ ability to reunite with family, make long-term plans, or feel emotionally secure. As Jasso [12] argues, such conditions produce sustained socio-economic and psychological stress, reinforcing broader evidence that immigration status is a key social determinant of mental health, linked to anxiety, depression, and chronic uncertainty [46, 47].

Financial sanctions against Iran further added another layer of structural vulnerability. Despite their legal presence in the U.S., many participants faced systemic barriers to accessing banking services, securing loans, and transferring funds between Iran and the U.S. Some faced sudden account closures, denied transactions, or excessive scrutiny – challenges that directly threatened their financial stability and deepened their sense of exclusion. The feeling of being “sanctioned,” despite having no political affiliations, exemplifies how sanctions perpetuate systemic patterns that individualize the consequences of policy, producing uncertainty and stress [13]. As one participant asked, “Why am I being sanctioned?” – a question that captured the emotional disorientation produced by depersonalized geopolitical targeting.

Despite these intersecting stressors, participants demonstrated remarkable psychological resilience. Many reframed their struggles as necessary sacrifices for long-term security, drawing on peer networks and a sense of community belonging as emotional support. This aligns with scholarship on immigrant coping, where resilience is seen as both structural navigation and emotional labor [48, 49]. However, as Willen [50] cautions, resilience under structural pressure often comes at a psychological cost, requiring sustained emotional effort to endure conditions of legal and social exclusion. Among highly educated Iranian immigrants, prior research shows that restrictive U.S. immigration policies function as social determinants of mental health, generating prolonged stress, anxiety, and social isolation [17]. This tension is echoed in Maghbouleh et al.’s [26] findings on the racialized positioning of Middle Eastern migrants – who, even as they navigate integration into the host country, continue to bear the toll of persistent state-sanctioned uncertainty.

## Strengths and Limitations

This study is among the first to examine how U.S. immigration policies affect the health and well-being of highly educated Iranian immigrants – a largely underexamined population that is both minoritized yet formally classified as White. While participants contribute significantly to U.S. society, they also face considerable challenges, including visa precarity, family separation, and financial exclusion. The study provides evidence of how nationality-based restrictions operate as psychosocial and material stressors, offering insight into the lived effects of policy on immigrant health. These findings can inform culturally sensitive and evidence-based interventions to improve support for Iranian immigrants.

Limitations include the potential influence of researcher positionality in theme selection and interpretation, despite effort to minimize bias through independent coding and peer validation. Additionally, the use of non-probability sampling limits generalizability. Participants’ accounts may also reflect retrospective bias, including memory lapses or social desirability effects. Nonetheless, the consistency of patterns across narratives strengthens the credibility and relevance of the findings.

## Conclusions and Recommendations

Our findings show that restrictive U.S. immigration policies, compounded by Iran-U.S. geopolitical tensions, impose sustained emotional, legal, and financial stress on highly educated Iranian immigrants. These pressures function as chronic stressors and are closely tied to mental health outcomes and barriers to integration. Participants’ migration decisions were driven by a complex mix of opportunity-seeking and structural push factors, including education, political repression, and gender-based constraints. However, arrival in the U.S. introduced new challenges – particularly legal precarity, family separation, and financial exclusion – that functioned as chronic stressors with direct psychosocial consequences such as anxiety, emotional exhaustion, and social isolation. These findings contribute to growing evidence that immigration systems, particularly when shaped by nationality-based restrictions, act as powerful social determinants of health.

To promote health equity, immigration policy should be decoupled from geopolitical conflict and offer transparent, stable visa pathways. Universities, advocacy organizations, and professional associations should expand support systems that not only seek to minimize legal and economic challenges such as visa navigation assistance programs, but also address the underlying emotional stressors that individuals experience through culturally responsive mental health care programs. Financial institutions must implement equitable practices to ensure legally present immigrants are not excluded from essential services due to nationality alone, which further exacerbate psychological well-being.

Future research should adopt health-centered and intersectional approaches to examine how immigration status, gender, and economic exclusion interact to shape well-being. Addressing these systemic barriers would allow host countries to protect immigrant health, promote social inclusion, and fully harness the contributions of highly educated migrants.

## Supplementary Information

Below is the link to the electronic supplementary material.

Supplementary Material 1

## Data Availability

Anonymized transcripts of the interviews for this study are available on request from the corresponding author.
