# Supplementary material for: Health Impacts of Migration and Policy on the Iranian Diaspora
Source: J Immigr Minor Health. 2026 Jan 7;28(4):935–44. doi: 10.1007/s10903-025-01844-1 (PMC13451459; doi:10.1007/s10903-025-01844-1)
Supplement: Supplementary file 1 [file 10903_2025_1844_MOESM1_ESM.docx]

**Supplementary File (Annex 1): Interview Protocol**

Thank you for participating in this research. Your input is valuable and greatly appreciated. We are not collecting any identifying information. If anything is shared by accident, it will be deleted from the transcript. This interview will be audio recorded for transcription purposes only; the video will not be saved and the recording will be deleted after transcription. You are welcome to turn off your camera at any time for your comfort.

**Interview Questions**

1. Can you tell me about your experience as an immigrant in the United States? Please feel free to share your background and what your life has been like as an immigrant here.

a. What factors have influenced your decision to pursue higher education in the US?

2. Have migration policies impacted your life in any way—either positively or negatively? Please share any experiences you feel comfortable discussing.
3. Can you describe your current support system? This could include friends, family, community networks, or institutional support.
4. Are you currently providing financial support to family members in your home country, or receiving financial support yourself—from family, educational institutions, or the government?
5. What has your experience been like in accessing financial services in the U.S., such as banking, credit, or loans?

b. If any, have you encountered any challenges related to your immigration status?

6. Do you have any suggestions for improving migration policies to better support the well-being of highly educated immigrants?
7. Is there anything else you’d like to share that we haven’t covered in this interview? Any additional thoughts or experiences you think are important.
